# Evaluating Machine Learning and Deep Learning models for predicting Wind Turbine power output from environmental factors

**DOI:** 10.1371/journal.pone.0317619

**Published:** 2025-01-23

**Authors:** Montaser Abdelsattar, Mohamed A. Ismeil, Karim Menoufi, Ahmed AbdelMoety, Ahmed Emad-Eldeen

**Affiliations:** 1 Department of Electrical Engineering, Faculty of Engineering, South Valley University, Qena, Egypt; 2 Electrical Engineering Department, Faculty of Engineering, King Khalid University, Abha, Saudi Arabia; 3 Renewable Energy Science and Engineering Department, Faculty of Postgraduate Studies for Advanced Sciences (PSAS), Beni-Suef University, Beni-Suef, Egypt; King Fahd University of Petroleum & Minerals, SAUDI ARABIA

## Abstract

This study presents a comprehensive comparative analysis of Machine Learning (ML) and Deep Learning (DL) models for predicting Wind Turbine (WT) power output based on environmental variables such as temperature, humidity, wind speed, and wind direction. Along with Artificial Neural Network (ANN), Long Short-Term Memory (LSTM), Recurrent Neural Network (RNN), and Convolutional Neural Network (CNN), the following ML models were looked at: Linear Regression (LR), Support Vector Regressor (SVR), Random Forest (RF), Extra Trees (ET), Adaptive Boosting (AdaBoost), Categorical Boosting (CatBoost), Extreme Gradient Boosting (XGBoost), and Light Gradient Boosting Machine (LightGBM). Using a dataset of 40,000 observations, the models were assessed based on R-squared, Mean Absolute Error (MAE), and Root Mean Square Error (RMSE). ET achieved the highest performance among ML models, with an R-squared value of 0.7231 and a RMSE of 0.1512. Among DL models, ANN demonstrated the best performance, achieving an R-squared value of 0.7248 and a RMSE of 0.1516. The results show that DL models, especially ANN, did slightly better than the best ML models. This means that they are better at modeling non-linear dependencies in multivariate data. Preprocessing techniques, including feature scaling and parameter tuning, improved model performance by enhancing data consistency and optimizing hyperparameters. When compared to previous benchmarks, the performance of both ANN and ET demonstrates significant predictive accuracy gains in WT power output forecasting. This study’s novelty lies in directly comparing a diverse range of ML and DL algorithms while highlighting the potential of advanced computational approaches for renewable energy optimization.

## 1. Introduction

### 1.1. Background information

#### 1.1.1. Renewable energy and wind power

Renewable energy and wind energy in particular are integral to the global shift toward environmentally friendly electricity generation [1, 2]. The governments and big energy producers advocate the use of wind together with solar and hydroelectric power as a way of accomplishing decarbonization in the energy sector. The incorporation of renewable energy systems into power systems entails unique characteristics that are very different from those associated with conventional power stations [3]. Despite these challenges, efforts are underway to develop technological tools and strategies that could improve the reliability and efficiency of power systems utilizing Renewable Energy Sources (RES) [4–6].

Forecasting and enhancing Wind Turbine (WT) efficacy is essential for the advancement of renewable energy. Wind and Photovoltaic (PV) solar energy constitute the cornerstone of renewable energy, offering clean, sustainable alternatives to fossil fuels that mitigate carbon emissions and advance global climate action goals [7, 8]. Accurate WT power production forecasts and PV system efficiency improve renewable energy dependability and efficiency, boosting energy security and sustainability [9, 10]. Machine Learning (ML) algorithms that anticipate WT production from environmental parameters improve energy management, resource allocation, and energy generation. This boosts renewable energy industry development and ensures a sustainable energy future. Improved predictive capacities may boost wind energy system efficiency, helping fulfill global sustainability goals and minimize non-renewable energy use [11].

Wind, solar, and hydropower are all crucial for reducing carbon emissions in the energy sector. But they don’t act like conventional power plants, so integrating them is difficult. There are critical issues to address, and consequently, it is important to focus on the creation of technological solutions and integration strategies [12].

The issue lies in the inherent fluctuations of solar and wind power, posing a challenge to the electrical grid. Nevertheless, this study can help us forecast and run the grid by exploring how these various sources complement one another. Therefore, it stresses the need for the integration of plentiful renewable sources [13].

Wind power technology needs to progress in order to transition away from fossil fuels. Looking forward to the present and future of wind energy conversion systems, which include mechanical and electrical components, there is an ongoing need for innovation to increase the efficiency and effectiveness of wind power generation [14].

Wind energy is a significant RES, which needs much technological development, both onshore and offshore, to meet increasing demand for the Carbon Neutral (CN) energy system for the support of RES. Wind power must resolve these issues to play the anticipated role in the future energy landscape [15].

Wind energy systems are being explored with increasing frequency using ML and optimization techniques to improve their efficiency and reliability. This is demonstrated by recent advancements in virtual wind speed prediction to suppress power fluctuations of micro-grids for stabilizing wind turbine output [16]. Furthermore, ML models have been shown to possess the potential for condition monitoring in wind turbines for fault detection and maintenance strategies that maximize their operation [17]. In addition, ML techniques have been successfully deployed for monitoring wind farm power curves [18]. Data-driven approaches to anomaly detection and to improve operational performance. In addition, eco-efficiency evaluations are supported by optimization models which are combined with novel ML approaches [19]. These innovations collectively underscore the transformative role of advanced technologies in addressing the challenges associated with renewable energy systems.

#### 1.1.2. Environmental variables

**Fig 1:**
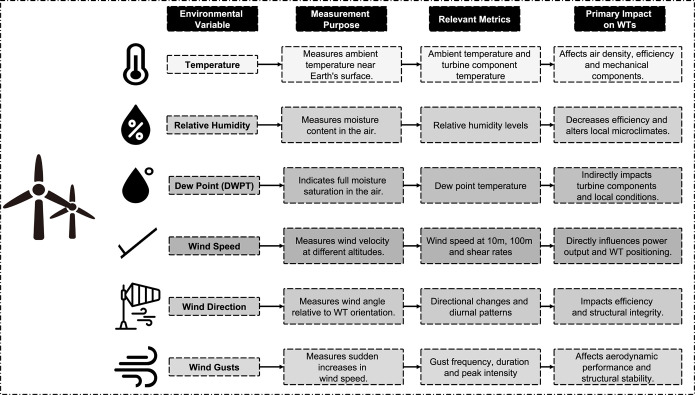
Environmental variables and their impact on WT power output.

Temperature, Relative Humidity (RH), Dew Point (DWPT), wind speed, wind direction, and wind gusts are the environmental variables that significantly affect the performance and efficiency of a WT. These factors each have an impact on turbine operation in unique ways, many of which present operational limits that require particular mitigation strategies to operate turbines efficiently. Air density and cooling requirements are a function of temperature and have significant impacts on the mechanical components of the turbine and overall energy efficiency, particularly when high temperatures cause problems like dust buildup and air filter clogging [20, 21]. In areas where humidity fluctuates, RH, which determines moisture in the air, is important, as higher RH is known to reduce energy efficiency and to increase WT maintenance demand especially in tropical climates [22–24]. The DWPT is the temperature at which air becomes fully saturated and is not necessarily conducive to condensing on turbine components but, when wet, requires the use of weather-resistant materials and covering components with protective enclosures [25]. Another important factor is the wind speed, which, as measured at different altitudes, has a direct impact on WT design, positioning, and energy output, primarily due to increasing focus to use higher wind speed as turbines become taller [26]. Both structural stability and energy production are affected by wind direction; therefore, during peak demand, directional changes lower turbine efficiency unless managed carefully [27]. Lastly, WTs are affected by wind gusts, which are sudden increases in wind speed that have an impact on both the WTs’ aerodynamic performance and their structural stability and which require advanced aerodynamic modeling to predict and manage these extreme conditions [28]. Fig 1 visually summarizes each of these variables and depicts icons, representing their measurement purpose, relevant metrics, and primary impact on WTs to improve turbine resilience and efficiency.

#### 1.1.3. Challenges in predicting WT power output

Forecasting The power output of WTs is very difficult to forecast due to the inherently erratic nature of the wind. For several recent studies have focused on several factors that make precise forecasts challenging, including unpredictable wind speed, the complexity of environmental interactions, and current predictive model limitations.

Short-term power forecasting is particularly hard because of the wind’s fluctuation and irregularity. A new approach using a composite Deep Learning (DL) based evolutionary method was proposed by Neshat et al. (2021) to produce more exact tracks of WT output forecasting. They show in their study [29] that WT output is difficult to model because wind speed is so unpredictable.

ML techniques are being used more and more to improve prediction models. Artificial intelligence was used to predict WT power accurately considering many environmental variables as described by Bhardwaj and co. (2022). Although these methods, however, will only be effective to the extent that there exists quality and quantity of data available for training [30].

The inherent unpredictability in wind power forecasting has been proposed to be solved based on probabilistic models. Ge, Zuo, and Tian (2020) proposed a probabilistic power curve model that outperforms deterministic models in the prediction of WTs power output. Such variations in the environment, particularly in the wind speed, must be considered [31].

However, when combining wind power onto the power grid, efforts to predict are further complicated. In the study of Rashid, Haider and Batunlu (2020), ML methods are used to predict WTs power generation. The authors in particular mentioned the challenges caused by unpredictable weather conditions, which directly influence wind speed and consequently power production [32].

#### 1.1.4. Machine learning and deep learning in wind power prediction

The effects of ML and DL on wind power prediction are used to optimize renewable energy resources. However, recent research points out that the use of other ML techniques, specifically DL algorithms, can result in high levels of accuracy and effectiveness in predicting wind energy [33]. From improving the ability to predict wind power output to precisely characterizing the turbulent and nonlinear nature of wind velocity, their research provides solutions to challenging issues.

Mohd and Singh (2023) investigated the potential use of Convolutional Neural Network (CNN), a kind of DL technique, for wind energy prediction. Initially, they focused on overcoming critical obstacles, such as weight initialization and the vanishing gradient problem, which are straightforward to overcome for precise forecasts [34].

Anushalini and Revathi (2024) examined ML algorithms for forecasting wind power output to mitigate the sporadic characteristics of RES. Anushalini and Revathi (2024) evaluated the forecast accuracy by utilizing historical wind data and several models to account for factors like speed, direction, and temperature. The best results were seen with Long Short-Term Memory (LSTM) and residual LSTM, which had mean absolute prediction accuracy of 0.0987 and 0.0958, respectively. This shows that they are effective at improving the management of renewable energy [35].

Karaman (2023) created a set of multi-objective models based on various ML techniques such as Artificial Neural Networks (ANNs), Recurrent Neural Networks (RNNs), CNNs, and LSTM networks to predict wind power. Karaman (2023) used two separate databases to predict wind power, demonstrating the effectiveness of LSTM in accurately forecasting wind power [36].

### 1.2. The motivation behind the study

The global energy sector would become adaptable and sustainable in the long run provided RES are adopted [37]. Among these sources of power, wind energy is remarkable because it can satisfy a significant part of future power needs [38]. However, the phenomenon of variability and unpredictability, associated with wind power generation, represent a major obstacle to its success in grid integration. Wind energy has the potential to be highly efficient; however, the lack of predictability in wind energy can lead to inefficiencies (distribution-wise), difficulties in managing demand response, and full utilization of wind energy’s potential.

In this research, the research investigates a set of ML algorithms, including Linear Regression (LR), Random Forest (RF), Extreme Gradient Boosting (XGBoost), Light Gradient Boosting Machine (LightGBM), Categorical Boosting (CatBoost), Adaptive Boosting (AdaBoost), Extra Trees (ET), and Support Vector Regressor (SVR) to evaluate their capacity to model and determine relationships between environmental variables and WT output. In addition, ANN, CNN, RNN, and Long Short Term Memory (LSTM) networks are evaluated as potential DL algorithms to learn complex dependencies in wind data. In studying these different approaches, the study aims to determine means of achieving the highest possible predictive accuracy in wind energy systems. This research is primarily driven by alignment of environmental sustainability objectives, advancement in ML and DL technology, and operational requirements of energy management in wind farms. This signifies a significant advancement in utilizing data and analytics to address the most urgent obstacles encountered by the renewable energy industry at present.

### 1.3. The problem statement

Wind energy is increasingly critical in the fight against climate change as countries push harder for renewable energy. But the erratic flow of wind speed and direction, and other factors that affect the output of the WT cannot be predicted with this characteristic. This variability complicates optimization of the energy produced and distributed, and it is difficult to maintain a stable and reliable power supply. However, the rapid fluctuation of wind energy makes it harder to manage the demand response in a way that will prevent wasted energy and insufficient supply during the high-demand time periods.

However, existing forecasting models often fail to adequately capture the highly nonlinear relationships that often exist among the environmental variables that affect power generation from WTs, e.g., wind speed, gusts, dewpoint, temperature, and RH. Further, the studies that exist are quite limited in regard to an overall comparison of ML & DL algorithms within the case of wind energy prediction. Even though there are numerous ML and DL methods, including LR, RF, Long Short Term Memory (LSTM) networks, and CNN, each has its own strengths and weaknesses, resulting in a gap in knowledge about the best suitable predictive models for this domain.

To bridge this gap, this study carries out a detailed evaluation and comparative study of eight ML algorithms, namely, LR, RF, XGBoost, LightGBM, CatBoost, AdaBoost, ET, and SVR, and four DL algorithms, namely, ANN, CNN, RNN, and LSTM Networks. This research aims to provide insights that are necessary to improve the accuracy of wind power predictions through an examination of each model’s performance in forecasting WT power output based on its specific environmental factors, to improve WT integration into the energy grid, and to help propel the global transition towards sustainable energy solutions.

### 1.4. Objectives of the research

**Fig 2:**
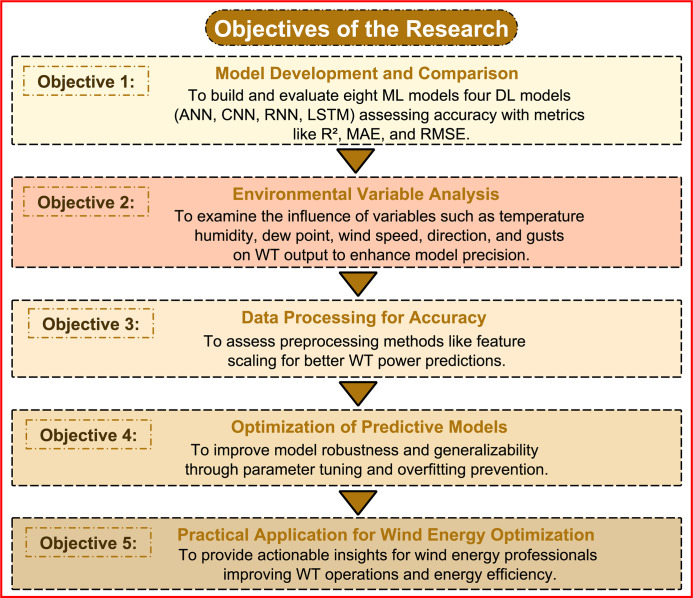
Research objectives for enhancing WT power output prediction using ML and DL models.

The principal objective of this research is to improve the predictive modeling of WT power production by utilizing various ML and DL algorithms. The explicit objectives are delineated in Fig 2, each addressing a distinct facet of model creation, data analysis, and practical implementation.

### 1.5. Contribution of the research

This research advances the renewable energy sector by offering a systematic and thorough assessment of ML and DL methodologies for forecasting WT power output based on environmental factors including temperature, humidity, wind speed, and wind direction. This study fills a notable gap in the literature by directly comparing different ML and DL models, offering insights into their suitability for handling complicated, multivariate data. The study highlights the importance of stringent preprocessing methods, including feature scaling and model parameter optimization, in enhancing predictive performance. This research provides a significant framework for utilizing advanced computational methods to tackle issues in wind power forecasting and optimize renewable energy resources through the exploration of varied algorithmic approaches and their applications to renewable energy systems. These contributions align with worldwide initiatives to enhance energy sustainability and reliability under climate change.

This paper is organized into several key sections to provide a clear and logical flow of the research paper. First of all, the research paper started with the ***introduction*** that presents the motivation, problem statement, and objectives, concluding with the contributions of this study. Secondly, the ***methodology*** describes the dataset, preprocessing steps, and the ML and DL models employed, along with the evaluation metrics used for performance comparison. Thirdly, the ***results and discussion*** section provides a detailed analysis of the predictive performance of the ML and DL models, supported by comprehensive visualizations and insights into the findings. Finally, the paper concludes with the ***conclusion***, which summarizes the key outcomes, emphasizes the implications for renewable energy systems, and delineates future research topics.

## 2. Methodology

### 2.1. Data presentation

**Fig 3:**
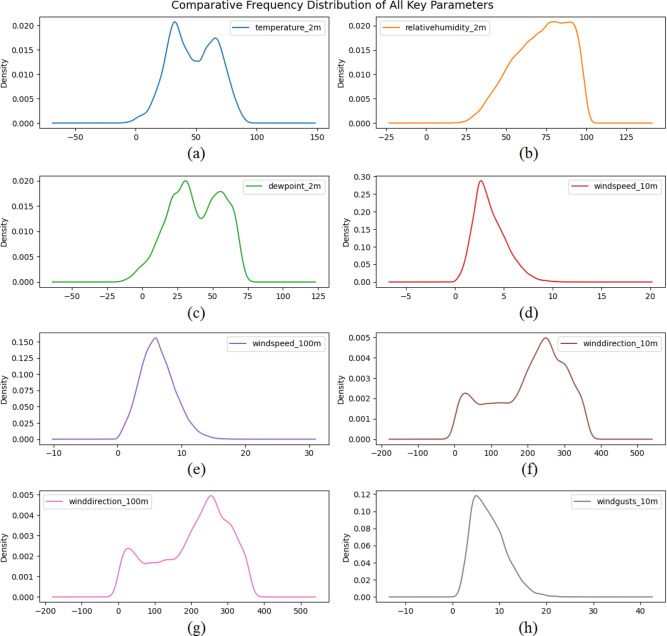
Density distributions of environmental parameters affecting WT energy output: (a) Temperature at 2m, (b) RH at 2m, (c) DWPT at 2m, (d) Wind speed at 10m, (e) Wind speed at 100m, (f) Wind direction at 10m, (g) Wind direction at 100m, (h) Wind gusts at 10m.

Evaluating the environmental factors that influence wind energy conversion is critical for maximizing WT efficiency. Fig 3 illustrates the density distributions of crucial environmental variables that play a key role in wind power generation. This includes Fig 3(A) displaying the temperature at a height of 2 meters, providing information about the thermal conditions; Fig 3(B) showing the RH at 2 meters, indicating the amount of moisture in the atmosphere; Fig 3(C) illustrating the DWPT at 2 meters, which is related to the likelihood of condensation; Fig 3(D) and 3(E) presenting the wind speed at 10 meters and 100 meters respectively, representing the amount of kinetic energy available for the turbine system at different altitudes; Fig 3(F) and 3(G) indicating the wind direction at 10 meters and 100 meters respectively, which impact the orientation and efficiency of the turbine; and Fig 3(H) highlighting the wind gusts at 10 meters, showcasing the variability in wind speed. A fundamental understanding of the distribution of the data is required before using ML and DL models to predict WT power output—and these visual depictions provide this understanding. These plots act as a good knowledge reserve for customizing the data preprocessing, which is expected to enhance the accuracy of the ML and DL algorithms used in this study. Density plots are also presented, emphasizing the diversity and variability of environmental parameters and the need for these to improve the predictive capability of ML and DL models. These data distributions also allow to identify trends and potential outliers as well as anomalies, and therefore help preprocessing and feature engineering steps to be adjusted to particular dataset characteristics.

The dataset used in this study consists of 40,000 observations that contain a full range of environmental variables that are necessary for predicting WT power output. The dataset utilized in our study is part of a public dataset licensed under CC0: Public Domain, with the specific portion used in the study detailed in the dataset availability section [39]. The descriptive statistics of these variables, such as temperature, RH, DWPT, wind speed (10m and 100m height), wind direction (10m and 100m height), wind gusts, and power output, are presented in Table 1. The range and distribution of each variable is summarized by these statistics, which provide values for the mean, standard deviation, minimum, maximum, and quartile points (25%, 50%, and 75%). The purpose of this statistical overview is to provide a basic understanding of the structure and variability of the dataset and the diversity of environmental conditions that ML and DL models will use to accurately predict turbine power output.

**Table 1:** Descriptive statistics of environmental variables in the dataset used for WT power prediction.

Feature	Mean	Std Dev	Min	25%	50%	75%	Max
**Temperature at 2m (°C)**	47.348	19.576	-14.400	31.700	46.400	64.100	94.100
**RH at 2m (%)**	71.912	16.942	18.000	59.000	74.000	86.000	100.000
**DWPT at 2m (°C)**	37.923	18.850	-17.100	23.600	37.100	54.200	76.300
**Wind Speed at 10m (m/s)**	3.603	1.648	0.000	2.420	3.310	4.600	13.450
**Wind Speed at 100m (m/s)**	6.298	2.678	0.100	4.400	6.090	7.990	20.650
**Wind Direction at 10m (°)**	203.639	96.985	1.000	131.000	225.000	278.000	360.000
**Wind Direction at 100m (°)**	203.313	98.580	0.000	129.000	226.000	279.000	360.000
**Wind Gusts at 10m (m/s)**	7.811	3.576	0.500	5.000	7.300	10.000	28.500
**Power Output (kW)**	0.408	0.287	0.000	0.153	0.352	0.660	0.991

**Fig 4:**
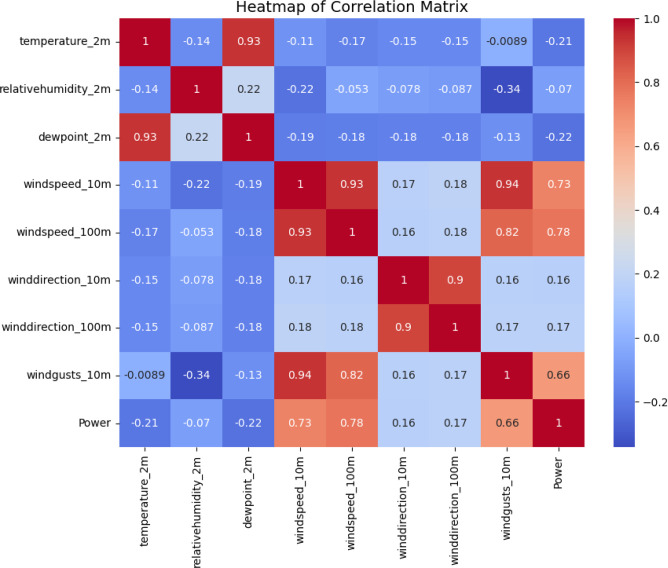
Heatmap of correlation matrix among environmental variables and power output.

Fig 4 illustrates the interconnections among environmental variables and their cumulative impact on WT power output, as part of the study’s comprehensive investigation. The study presents the correlation matrix as a color-coded heatmap, illustrating the strength and direction of relationships between various weather variables, such as temperature at 2 meters, RH at 2 meters, DWPT temperature at 2 meters, wind speeds at 10 and 100 meters, wind directions at 10 and 100 meters, wind gusts at 10 meters, and the resulting power output. Color intensity is linked to the strength of the correlation coefficients; more negative correlations are shown by cooler hues, whereas warmer hues imply more positive correlations. In addition to highlighting important relationships between certain environmental factors and electricity generation, this graphic analysis also shows the possibility of multicollinearity among the predictors. Important details for the upcoming modeling stage are shown in Fig 4. It helps choose attributes for the ML and DL algorithms’ input and makes it easier to identify the factors that have the biggest effects on WT efficiency. An essential tool for understanding the complex interactions between environmental factors affecting wind energy output is a correlation heatmap.

This research examines the complementary roles of forecasting and data augmentation in improving the predicted accuracy of WT power production. Forecasting emphasizes the application of ML and DL models to anticipate WT power output by analyzing intricate correlations among environmental variables, including wind speed, temperature, and humidity. Data augmentation is an essential preprocessing step that enhances data quality and prepares the model. Methods like feature scaling, addressing missing data, and sequence generation are employed to improve model efficacy, especially for models that are sensitive to feature ranges, like SVR, or those that necessitate temporal data, such as LSTM networks. The research indicates that ensemble ML models, such as ET, are superior at capturing feature interactions and variance, whereas DL methods, like ANN, excel in identifying nonlinear dependencies in multivariate data. These techniques together affirm the efficacy of the selected methodology, wherein rigorous data preprocessing and augmentation substantially improve the predictive performance of the forecasting models, guaranteeing elevated accuracy and generalizability in WT power prediction.

### 2.2. Machine learning algorithms

**Fig 5:**
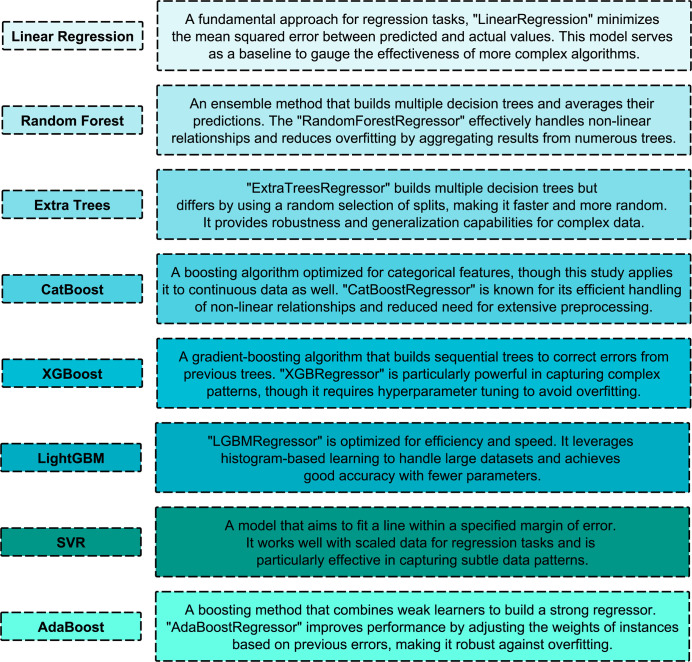
Overview of ML model selection and training for WT power output prediction.

In this study, several ML algorithms are applied to predict WT power output using environmental variables, each of which is chosen for its specific advantage in regression tasks. It contains features such as temperature, humidity, wind speed, wind direction at various altitudes and a target variable, ‘Power’ which is the output of the turbine. First, the dataset goes through preprocessing, where the ‘Time’ column is converted to a ‘datetime’ format. The data is split into training and testing sets with an 80–20 ratio to be able to evaluate unbiasedly on unseen data. For models sensitive to feature scales (e.g., SVR, LR), features are standardized using ‘StandardScaler’. This allows scale-sensitive models to interpret the data accurately. The analysis includes eight ML algorithms, as illustrated in Fig 5, starting with LR, RF, ET, CatBoost, XGBoost, LightGBM, SVR, and AdaBoost, which were used.

A custom ‘train_and_evaluate’ function trains each model on training data and generates predictions on the test set. Model performance is evaluated using three primary metrics: Mean Absolute Error (MAE), Root Mean Square Error (RMSE), and R^2^. Higher values for R^2^ indicate a better fit, as they indicate the proportion of variance in the target variable the model explains. Both MAE and RMSE measure the accuracy of prediction, with lower values for both indicating higher accuracy. MAE is the average magnitude of errors in predictions and RMSE is the standard deviation of prediction errors.

Each model’s result is compiled as a ‘DataFrame’ and then saved to a CSV file for reference. Visualizations are used to interpret these results further: R^2^, MAE, and RMSE are bar charted for each model, which favors high results for R^2^ and low results for MAE and RMSE. A further heatmap of correlations also including the ‘Power’ variable shows feature interactions and aids the interpretation of the variable importance. A visual benchmark of model accuracy is provided in the form of scatter plots of actual versus predicted values for each model, for which the proximity of the data points to a diagonal reference line indicates prediction quality. These visualizations enable comprehensive analysis, helping to determine the models most effective in predicting WT power output based on environmental conditions.

Table 2 provides a detailed summary of the ML models employed in this study to forecast WT power production. It emphasizes essential elements such as the principal parameters for each model, the necessity of feature scaling, the model’s classification as a baseline or advanced technique, and certain prerequisites for training data. This overview elucidates the configuration of each model and its distinct contributions to the regression tasks in this investigation.

**Table 2:** Summary of ML models: Key parameters, scaling requirements, baseline usage, and training data needs.

Model	Key Parameters	Scaling Requirement	Baseline Usage	Training Data Requirement
LR	No parameters needed for basic implementation	Required	Baseline	Full dataset
RF Regressor	random_state = 42 (ensures reproducibility)	Not Required	Advanced	Bagging over subsets
XGBoost Regressor	random_state = 42, verbosity = 0 (verbosity off)	Not Required	Advanced	Full dataset
LightGBM Regressor	random_state = 42	Not Required	Advanced	Full dataset
CatBoost Regressor	random_state = 42, verbose = 0 (verbosity off)	Not Required	Advanced	Full dataset
AdaBoost Regressor	random_state = 42	Not Required	Intermediate	Full dataset
ET Regressor	random_state = 42	Not Required	Advanced	Bagging over subsets
SVR	Uses scaled data, kernel and regularization tunable	Required	Intermediate	Full dataset

**Fig 6:**
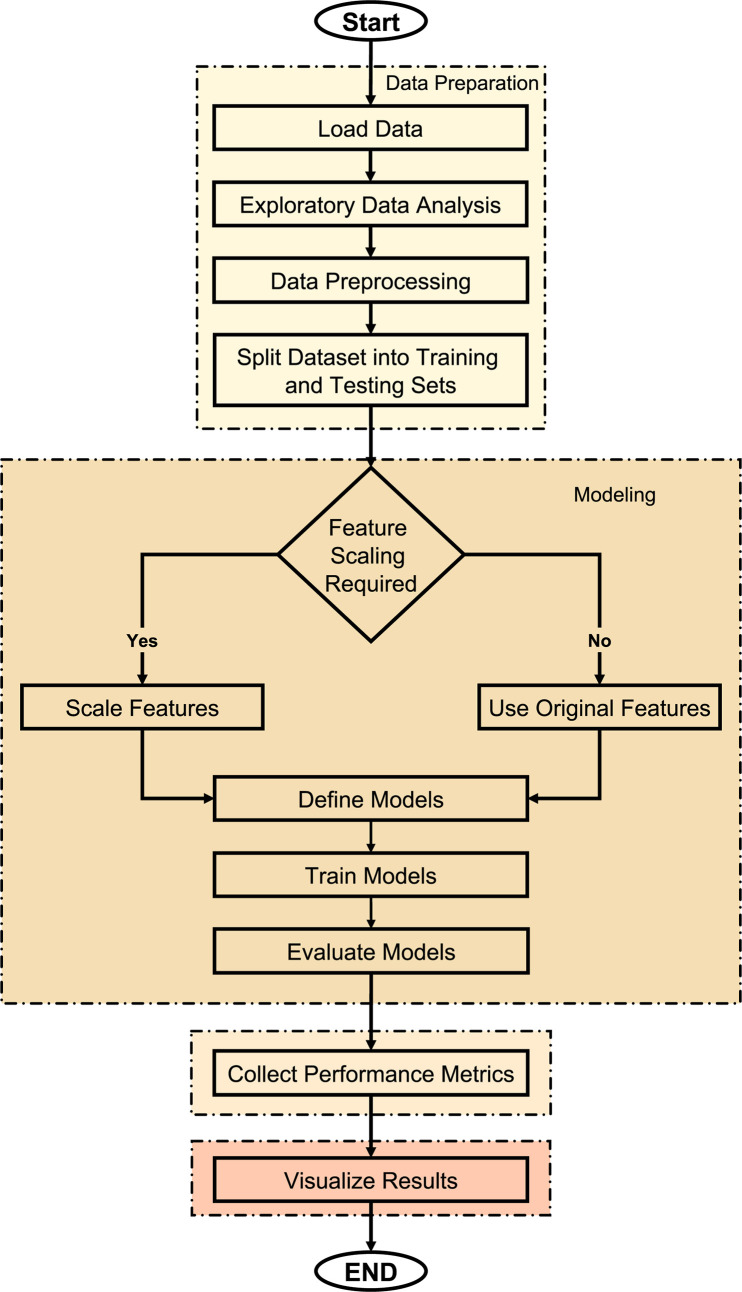
Flowchart of ML methodology for predicting WT power output.

Fig 6 illustrates the progression of the ML process, detailing the phases from data preparation to model evaluation in this study. Initially, data is imported, followed by the execution of Exploratory Data Analysis (EDA) to identify any trends, patterns, or outliers present in the dataset. After EDA, data preprocessing is done, which includes steps like handling missing values and encoding categorical variables in order to clean and prepare the data. It is then split into training and testing sets for unbiased model evaluation. To continue the workflow, it splits, checks if feature scaling is required, and then it splits once more. Features are standardized (if needed) to follow models, e.g. SVR and LR, that are sensitive to scale. The following stage is the definition of ML models, including ensemble techniques like ET and RF, boosting algorithms like CatBoost, XGBoost, LightGBM, AdaBoost, and traditional models like LR. The models are then trained on the training dataset, and performance is evaluated using R^2^, MAE, and RMSE on the testing set. Performance metrics are collected, and results are visualized for comparative analysis to gain insights into which model is most accurate in predicting WT power output as a function of environmental variables. This flowchart is a structured way to do model selection and evaluation for multiple ML models.

### 2.3. Deep learning algorithms

This study employs DL algorithms to uncover associative, non-linear relationships between forecasting WT power output and environmental data. Unlike traditional ML methods, DL models excel at detecting intricate patterns in such data and are thus optimal for time series data, such as wind and weather measurements. DL models used here are LSTM, ANN, RNN, and CNN. The benefit of each model is unique: LSTMs and RNNs are designed to capture temporal dependencies essential for forecasting a trend in sequential data, and CNNs are good at recognizing local patterns in the series of data. In this section, the architecture, training, and assessment of each DL model are delineated, which, in turn, compares the effectiveness in predicting the precise power production of WT with high accuracy.

The attributes of each DL model in this study used for forecasting WT power production are delineated in Table 3. This table compares the models with regard to their fundamental layers and characteristics, suitability for sequential data, training complexity, scaling requirements, and important considerations. ANN and CNN are better suited for general regression applications, whereas LSTM and RNN models are more adept at handling sequential data as they can capture temporal dependencies quite well. Different quantities of units, filters, and activation functions in each model make each of the model’s performance and optimization different during training.

**Table 3:** Comparison of DL models used in the study for predicting WT power output.

Model	Key Layers & Features	Suitable for Sequential Data	Training Complexity	Requires Scaling	Key Parameters
LSTM	LSTM layer, Dense output	Yes	High	Yes	Units in LSTM layer, activation
ANN	Dense (fully connected) layers	No	Medium	Yes	Number of Dense layers, activation
RNN	SimpleRNN layer, Dense output	Yes	Medium	Yes	Units in RNN layer, activation
CNN	Conv1D, MaxPooling1D, Flatten, Dense	No	Medium-High	Yes	Filters, kernel size, pooling size

**Fig 7:**
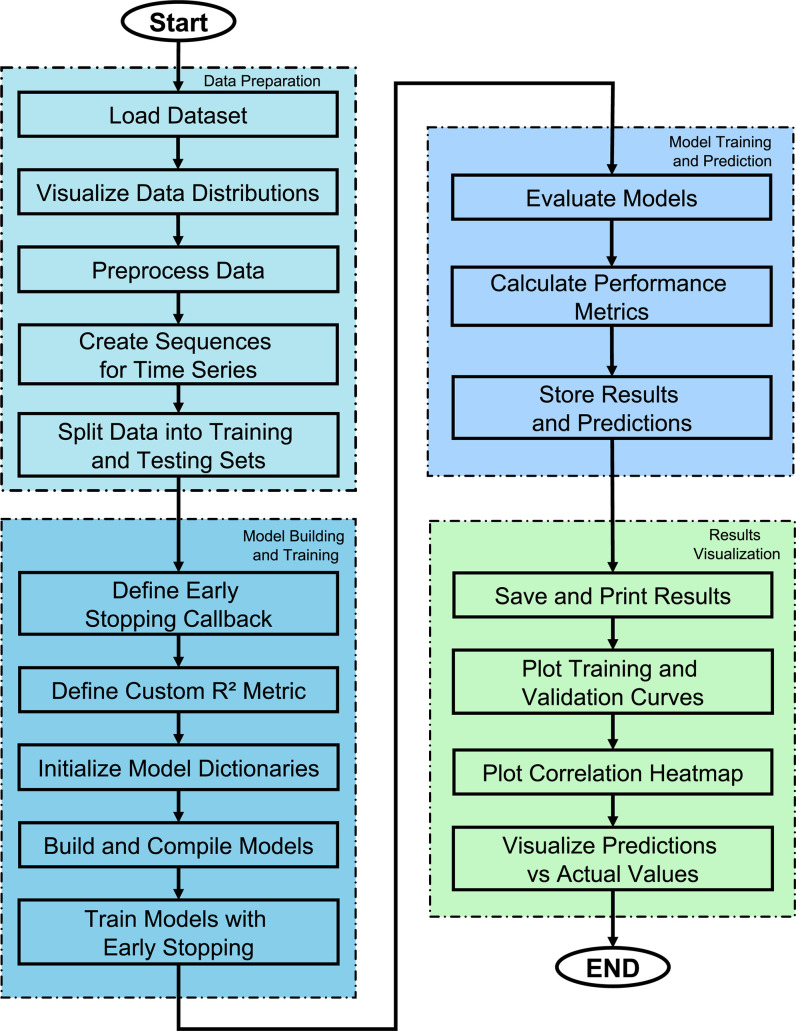
DL workflow flowchart for predicting WT power output from environmental variables.

The flowchart in Fig 7 illustrates the DL technology employed to forecast WT power output depending on environmental data. The procedure is segmented into four principal phases: Data preparation, Model construction and training, model assessment, and results visualization.

The Data preparation phase commences the workflow, during which the dataset is loaded and preliminary data exploration is conducted through the visualization of important parameter distributions. Subsequently, data preprocessing is performed to execute requisite modifications. As this is a time-series analysis, sequences are generated to encapsulate temporal dependencies, followed by the division of the dataset into training and testing sets to enable model evaluation on novel data.

During the model building and training phase, early stopping callbacks are established to avert overfitting by ceasing training when validation loss ceases to improve. A bespoke R^2^ statistic is employed to assess model performance, and dictionaries are established to categorize the models and their corresponding histories. Each DL model—LSTM, ANN, RNN, and CNN—is developed, built, and trained on the predefined sequences with early stopping activated to enhance performance.

In the model evaluation phase, each trained model is assessed on the test set to compute performance measures such as R^2^, MAE, and RMSE, offering insights into the prediction accuracy of each model. Results and forecasts are archived for subsequent analysis.

Results visualization follows and saves metrics of performance of each model into the results folder and plots training and validation curves to observe how each model learns. A correlation heatmap and scatter plots of predictions vs. actual values are provided for each model, enhancing understanding of the modeling accuracy and feature relationships and helping to understand how each model is effective at predicting WT power output from environmental variables. Such an approach of structuring the study was appropriate for conducting a detailed comparative analysis of DL techniques.

### 2.4. Evaluation metrics

It is essential to utilize precise performance and accuracy metrics when comparing multiple ML and DL methods for predicting WT power output using environmental factors. ML and DL algorithms are evaluated using three main metrics: RMSE, MAE, and R^2^. Each of these metrics, ranging from the degree of variance accounting to the average size of prediction errors, offers valuable insights into various aspects of model performance.

#### *R* ^2^

The *R*^2^ metric measures how much of the variance in the dependent variable the independent variables can correctly forecast. The metric gives an indication of how well the model predicts the target variable; a value nearer to 1 indicates a model that does so. One important measure to evaluate the model’s capacity to explain the variation in WT power output depending on environmental factors is *R*^2^. Eq (1) defines the formula for computing *R*^2^, which quantifies the percentage of the variance in the target variable that the model explains in relation to the total variance. This gives a sense of how well the model represents the intrinsic variability in the target variable [39].

#### MAE

It measures the mean absolute mistakes of a certain set of predictions irrespective of their direction. The measure is calculated in this study by averaging the absolute differences between the anticipated and observed values providing a direct measure of prediction accuracy. The lower the MAE number, the more greatly does it pull the model to become more precise in its ability to predict power output. As a result, MAE is a critical indicator to determine the performance of different models within a uniform scale. The computation of MAE as presented in Eq (2) is done by aggregating absolute errors of all these set of forecasts to produce an average error magnitude [40].

#### RMSE

Is a quadratic scoring rule which quantifies the average magnitude of the error. This is the square root of the mean squared deviations between predicted value and the observation. The RMSE will first square the errors and then set them to take the average, which gives considerably more of a weight to large errors. However, in cases where significant errors are particularly undesirable, it is where RMSE is best. The smaller the RMSE, the better the model’s performance. The Eq (3) describes the formula of RMSE in detail, which explains how the RMSE itself isn’t an exact measure of predictive accuracy due to its imposition of harsher penalties on large errors [41].

These metrics were calculated on the test data for each model to provide a robust evaluation of model performance on unseen data. The results allow for a direct comparison between traditional ML models and DL models, highlighting the strengths of each approach in handling complex, non-linear relationships inherent in environmental data for WT power prediction. These evaluation metrics have also been used in this study, as well as in a study that applied ML and DL for prediction, as indicated in [42].


$$R^{2}=1-\frac{\sum_{i=1}^{n}{\left(y_{i}-{\hat{y}}_{i}\right)}^{2}}{\sum_{i=1}^{n}{\left(y_{i}-\bar{y}\right)}^{2}}$$ (1)

$$MAE=\frac{1}{n}\sum_{i=1}^{n}\left|y_{i}-{\hat{y}}_{i}\right|$$ (2)

$$RMSE=\sqrt{\frac{1}{n}\sum_{i=1}^{n}{\left(y_{i}-{\hat{y}}_{i}\right)}^{2}}$$ (3)

The three equations provide metrics to evaluate model performance in predicting WT power output:

- Eq (1) R^2^ measures the fraction of variance in the observed data accounted for by the model, with values approaching 1 signifying superior performance.
- Eq (2) MAE provides a direct assessment of predictive accuracy, where diminished values signify enhanced precision.
- RMSE in Eq (3) takes the square root of mean squared differences, which means it is suitable for evaluating models in which the reduction of large errors is necessary, as it penalizes large errors.

The three metrics in the previous equations represent a complete performance analysis (accuracy and reliability prediction) of the model.

## 3. Results and discussion

### 3.1. Machie learning results

In this section, ML models such as AdaBoost, LR, SVR, LightGBM, XGBoost, CatBoost, RF and ET are analyzed in depth to predict WT power output as a function of environmental variables.

In Table 4 a comparative analysis of various ML models used to predict WT power output given environmental variables is presented. The models are ranked from low to high R^2^ value to give us a sense of how predictive they are. The models evaluated are ranked based on R^2^, ranging from 0.6147 for AdaBoost, least fit for this dataset, to 0.7231 for ET, highest predictive accuracy.

**Table 4:** Performance comparison of ML models for predicting WT power output.

Model	R^2^	MAE	RMSE
AdaBoost	0.6147	0.1458	0.1784
LR	0.6175	0.1406	0.1777
SVR	0.6922	0.1201	0.1594
LightGBM	0.7026	0.1195	0.1567
XGBoost	0.7062	0.1180	0.1557
CatBoost	0.7120	0.1170	0.1542
RF	0.7185	0.1142	0.1525
ET	0.7231	0.1132	0.1512

In addition, Table 4 presents MAE and RMSE for each model to show prediction error characteristics in detail. These models (ET and RF) have R^2^ values (0.7231 and 0.7185 respectively) that are high, as well as low MAE and RMSE, indicating consistence in minimizing error. On the other hand, models with lower R^2^, such as AdaBoost and LR, have higher error metrics which indicate more fragile predictive abilities. As shown in Table 4, ensemble methods like ET and RF are more capable of capturing the relationship between WT power output and environmental variables than other methods such as AdaBoost and LR.

**Fig 8:**
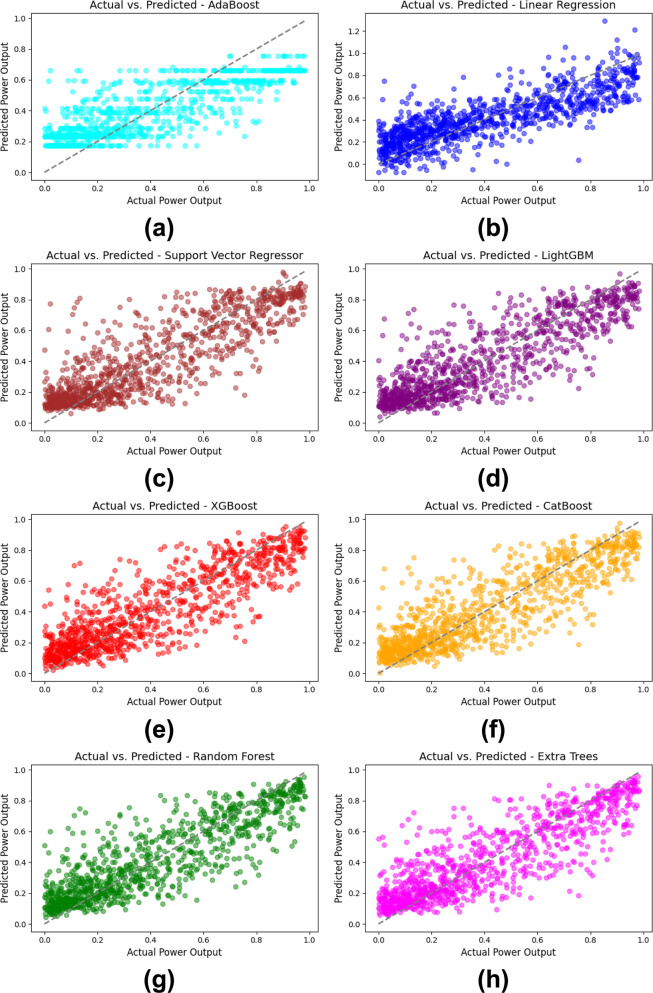
Comparative Actual vs. Predicted Power output by ML model for the first 1000 observations: (a) AdaBoost, (b) LR, (c) SVR, (d) LightGBM, (e) XGBoost, (f) CatBoost, (g) RF, (h) ET.

Fig 8 provides a comparative visualization of actual versus predicted power output for the first 1000 observations across different ML models. Fig 8(A)–8(H) are subplots representing each model, enabling one to visually compare the accuracy of each model. The spread of the predicted values around the actual values is higher in AdaBoost model as depicted in Fig 8(A) especially at lower output levels, and this suggests that the model makes less accurate predictions as compared to other models. Fig 8(B) for LR shows an enhancement; there is a more evident positive slope, but there is still fluctuation and oscillation from the perfect line. The SVR in Fig 8(C) has a better clustering about the diagonal line than the other two models.

There is a closer to diagonal distribution of points in Fig 8(D) and 8(E) related to LightGBM and XGBoost, respectively, which means that the algorithms are better in terms of the correlation between actual and predicted power output. As with Fig 8(F), CatBoost also remains aligned along the diagonal in a consistent manner. Fig 8(G) and 8(H) which depict RF and ET show the best predictive performance with most of the data points lying nearer to the ideal diagonal line. This means that these ensemble models, especially ET are more capable of predicting power output as depicted by the least spread and low error. As illustrated in Fig 8, ET and RF which are ensemble methods, outcompete simple methods like AdaBoost and LR in accurately predicting the WT power output.

**Fig 9:**
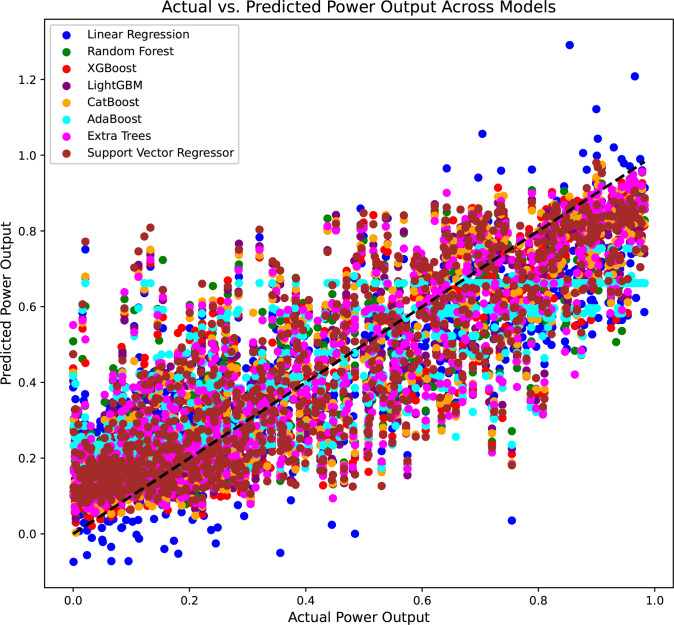
Aggregated actual vs. predicted power output across all ML models.

A consolidated assessment of the predictive efficacy of all ML models used in the study to predict WT power generation is presented in Fig 9. The study depicts a scatter plot of actual versus expected power output values for each model plotted for all models, each model represented by a unique color. Fig 9 features a reference diagonal line that signifies the ideal alignment where anticipated values correspond precisely with actual values.

The concentrated clustering along the diagonal line signifies that the majority of models successfully approximated the actual power output with considerable precision. The measured dispersion around this line indicates differing levels of predictive accuracy among the models. Significantly, certain spots diverge markedly from the line, especially at elevated production levels, highlighting regions where models had difficulties in achieving accurate predictions.

To conduct a thorough assessment, metrics like MAE and RMSE were computed for the complete dataset of 40,000 observations. These measurements offer a comprehensive evaluation of each model’s performance, corroborating information from the scatter plot regarding which models most effectively represent the underlying data patterns.

**Fig 10:**
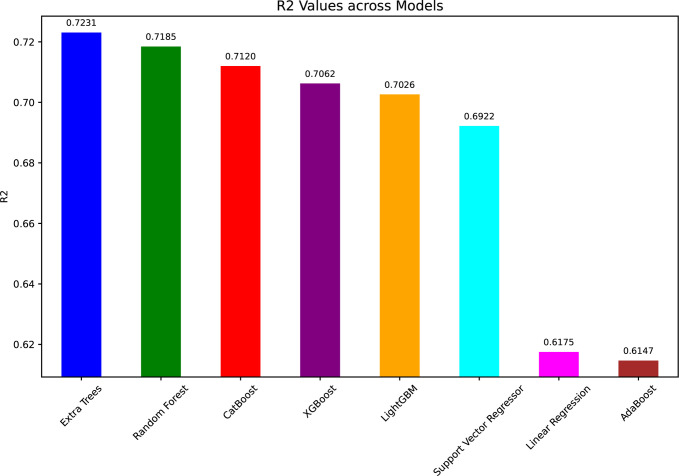
R^2^ values for ML models in predicting WT power output.

Fig 10 (R^2^ values) shows the explained variance of the models; ET had the highest R^2^ value of 0.7231, followed by RF (0.7185) and CatBoost (0.7120). This suggests that these models are better suited to describe the variability in the WT power output data. Contrary to that, LR and AdaBoost achieved a lower performance with R^2^ values of 0.6175 and 0.6147, respectively.

**Fig 11:**
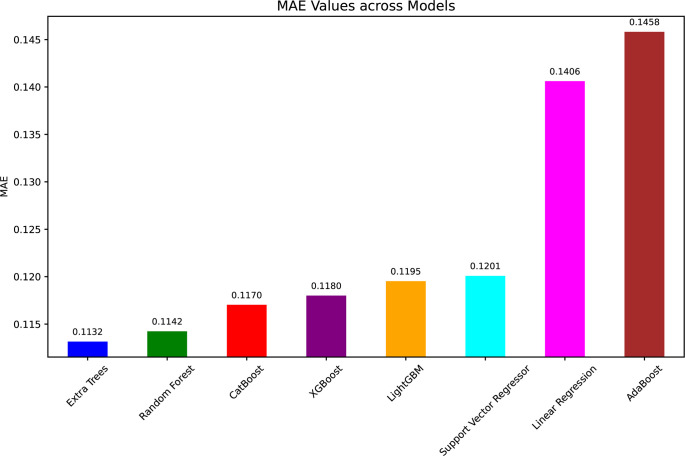
MAE values for ML models in WT power prediction.

Fig 11 (MAE values) agrees with these findings as ET had the lowest MAE of 0.1132 followed by RF and CatBoost. The error values of 0.1458 for AdaBoost and also 0.1406 for LR were the highest for these two algorithms and this can be taken as a sign of weakness for the two algorithms to give accurate predictions in this case.

In summary, tree based ensemble methods, particularly ET and RF, have performed better than other models in all evaluation metrics. Because of this, they were the best ML models for estimating WT power output from environmental variables. This performance ranking is consistent with Table 4 and confirms that these methods are suitable for predictive modeling in renewable energy applications.

**Fig 12:**
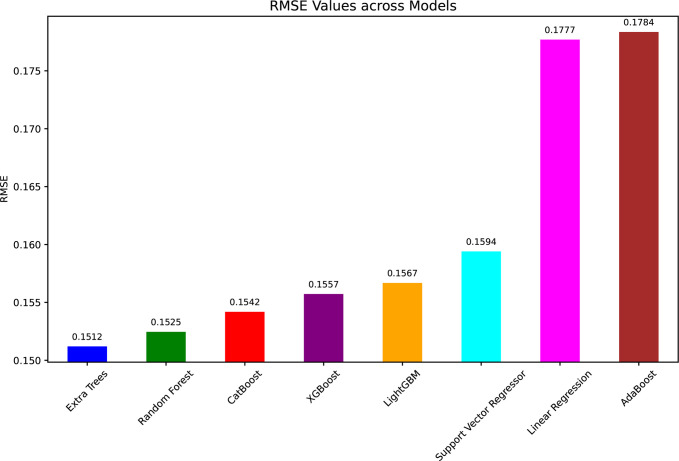
RMSE values for ML models in WT power prediction.

In Fig 12 (RMSE values), ET had the lowest RMSE of 0.1512 very close followed by the RF (0.1525) and CatBoost (0.1542). However, the RMSE values of LR and AdaBoost models were the highest, 0.1777 and 0.1784 respectively. This demonstrated that they weren’t all that good at reducing prediction error compared to tree based ensemble approaches.

### 3.2. Deep learning results

This section provides a comprehensive examination of four DL models—CNN, LSTM, RNN, and ANN—regarding their efficacy in predicting WT power production, emphasizing critical metrics such as R^2^, MAE, and RMSE to assess model accuracy and generalization.

Table 5 presents a comparative examination of four DL models—CNN, LSTM, RNN, and ANN—in forecasting WT power output, assessed using R^2^, MAE, and RMSE metrics. Table 5 demonstrates that the ANN model attained the greatest R^2^ value of 0.7248, signifying its exceptional accuracy in representing the variance in wind power output. The ANN model demonstrated the lowest error rates, with an MAE of 0.1136 and an RMSE of 0.1516, highlighting its efficacy in reducing prediction errors. On the opposite side, the CNN model had the lowest R^2^ score of 0.6297, yet it had the highest MAE and RMSE and therefore was the least effective of all models for this specific prediction task. The accuracy and error measures for the RNN model were compared with those of LSTM, and the RNN model performed better than LSTM by a small margin. Consequently, Table 5 is an illustration of the robustness and the generalization capabilities of the ANN, which allows us to conclude that this model is the most promising architecture among all those investigated in this study.

**Table 5:** Performance comparison of DL models for predicting WT power output.

Model	R^2^	MAE	RMSE
**CNN**	0.6297	0.1394	0.1759
**LSTM**	0.6346	0.1364	0.1747
**RNN**	0.6551	0.1330	0.1698
**ANN**	0.7248	0.1136	0.1516

**Fig 13:**
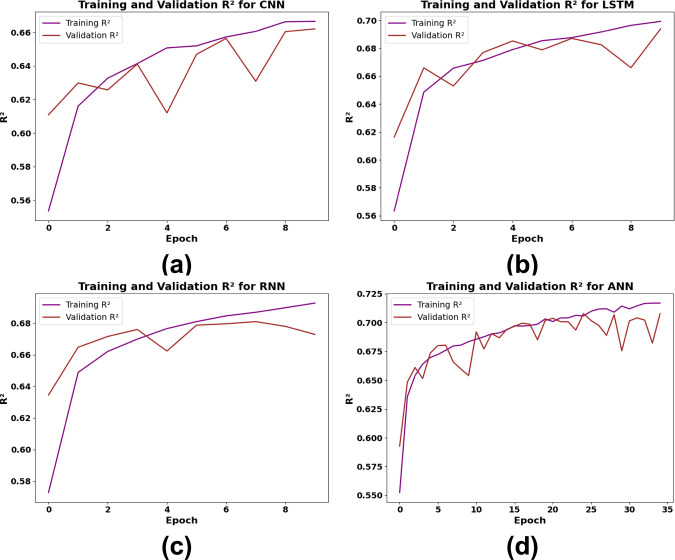
Training and validation R^2^ performance of DL models (a) CNN, (b) LSTM, (c) RNN, (d) ANN.

Fig 13 Four DL models—CNN, LSTM, RNN, and ANN—are shown in Fig 13 together with their R^2^ performance trends over training epochs, emphasizing both training and validation R^2^ values. The CNN model exhibits a slow rise in training R^2^ with discernible variations in validation R^2^ in Fig 13(A), indicating some challenges with generalizing to new data. The performance of the LSTM model is shown in Fig 13(B), which again shows quick early improvement in training and validation R^2^ but shows significant variability in validation scores, suggesting possible overfitting. The RNN model, as illustrated in Fig 13(C), exhibits a modest capacity for generalization, achieving a rather steady and constant improvement in training R^2^ with very slight variations in validation performance. The ANN model, which has the longest training period and achieves the best R^2^ values for both training and validation with the fewest variations in the validation curve, is finally shown in Fig 13(D). This stability shows that the ANN model outperforms the other models in terms of accuracy and consistency across epochs, indicating that it not only learns from the data but also generalizes well.

**Fig 14:**
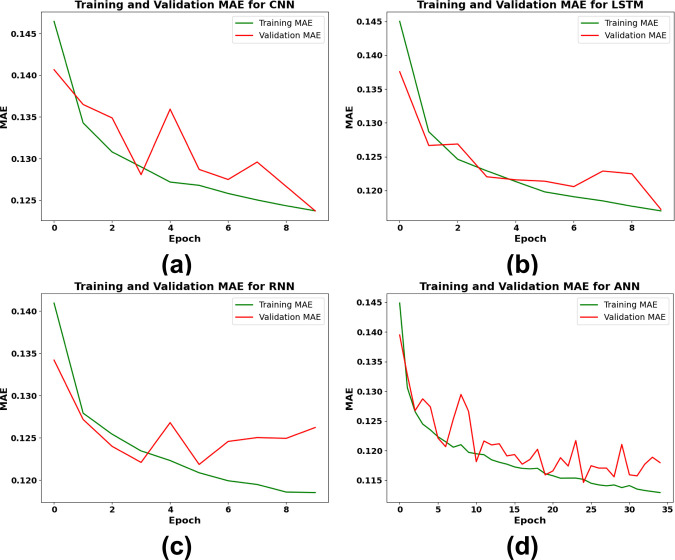
Training and validation MAE performance of DL models (a) CNN, (b) LSTM, (c) RNN, (d) ANN.

Fig 14 illustrates the trends of MAE for training and validation across epochs for each DL model—CNN, LSTM, RNN, and ANN—over the course of 100 epochs, incorporating an early stopping function. The data presented offers valuable insights into the models’ error reduction and generalization capabilities.

The CNN model shows a declining trend in both training and validation MAE in Fig 14(A), with training MAE at the last epoch at around 0.123. Further epochs show substantial generalizations but some variability on unknown data, as the validation MAE also declines but varies between 0.130 and 0.125. Table 5 summarizes CNN’s final MAE, which was 0.1394, indicating that it performed rather poorly among the models. Fig 14(B) shows the MAE trends for the LSTM model. In the first few epochs, both the training and validation MAE drop down quickly, approaching 0.118. With validation MAE stabilizing around 0.118 in subsequent epochs, the curves are quite similar, suggesting that LSTM efficiently reduces error while generalizing rather well. According to Table 5, LSTM’s final MAE is 0.1364, which places it just ahead of CNN but still below ANN and RNN. The RNN model’s MAE performance is shown in Fig 14(C), where training MAE reduces gradually and reaches around 0.117 by the last epoch. Although it shows some oscillations around 0.125, the validation MAE curve is quite steady, indicating acceptable generalization. RNN outperforms CNN and LSTM in handling the temporal features of the data, as evidenced by its final MAE of 0.1330, which is shown in Table 5. The training MAE of the ANN model drops to about 0.113 by the 35th epoch, displaying the lowest MAE values in Fig 14(D). The validation MAE follows a similar trend but with minor fluctuations and stabilizes at 0.113. MAE of training and validation are very close, including that ANN has a better generalization and lower error rate on unknown data. Consequently, as indicated in Table 5, the final MAE for ANN model was 0.1136 which is the most accurate model and showed its reliability and robustness for forecasting WT power production.

**Fig 15:**
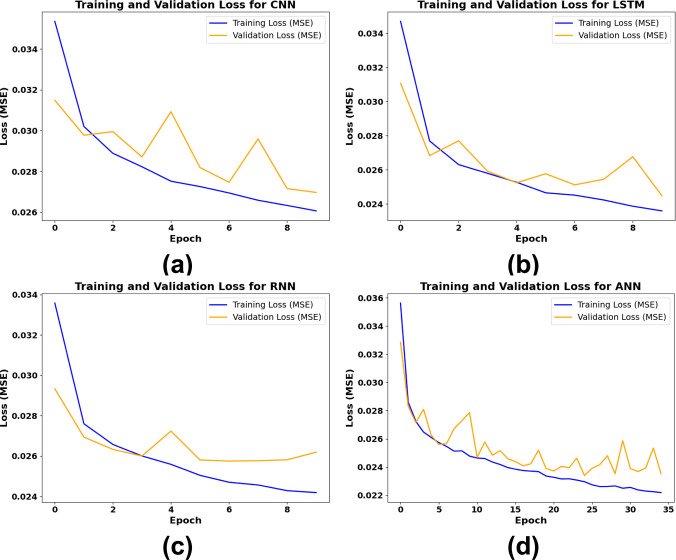
Training and validation loss performance of DL models (a) CNN, (b) LSTM, (c) RNN, (d) ANN.

Fig 15 shows the training and validation loss trends for CNN, LSTM, RNN, and ANN models over epochs, highlighting each model’s ability to generalize. In Fig 15(A), CNN achieves a steady reduction in training loss but shows fluctuating validation loss, indicating moderate generalization. Fig 15(B) for LSTM displays a close alignment between training and validation loss, stabilizing around 0.025, though with occasional validation spikes. Fig 15(C) illustrates RNN’s stable training loss decrease and consistent validation performance, reflecting excellent generalization. Finally, Fig 15(D) reveals that ANN reaches the lowest training and validation loss, with smooth, closely aligned curves, confirming its robust performance and minimal overfitting.

**Fig 16:**
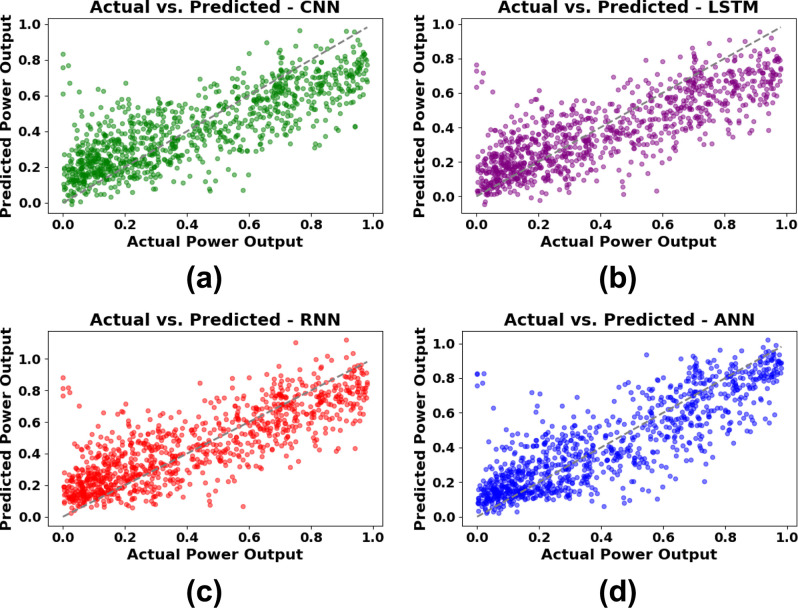
Predicted vs. Actual Power output for DL models on a sample of 1000 data points (a) CNN, (b) LSTM, (c) RNN, (d) ANN.

Fig 16 shows the comparison between the expected and actual power output for a group of 1000 data points, which tests how well CNN, LSTM, RNN, and ANN models work. In Fig 16(A), the CNN model has a moderate correlation with the real data, while there is significant variability, particularly at elevated output levels. Fig 16(B) for LSTM exhibits a comparable distribution, with predictions congregating along the trend line while demonstrating discrepancies in properly identifying elevated outputs. Fig 16(C) depicts the RNN outcomes, demonstrating that predictions roughly approximate the actual values with a marginal enhancement in alignment, signifying satisfactory performance. The ANN model that corresponds most accurately with the actual output values is shown in Fig 16(D), where data points are more densely packed along the trend line, thereby again confirming the superiority of ANN in predicting power output as compared with the other models studied.

**Fig 17:**
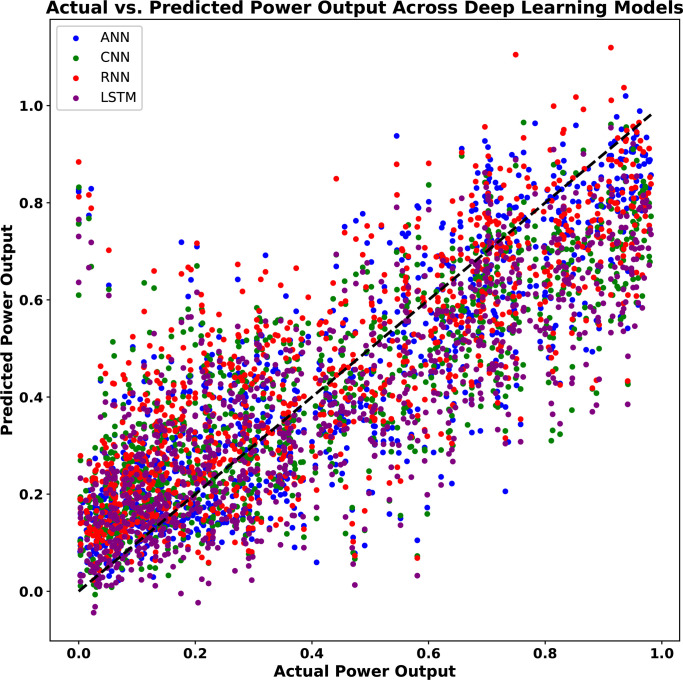
Aggregated actual vs. predicted power output across all DL models.

Fig 17 shows the aggregated actual versus predicted power output across 1000 observations from 40,000 sets for the DL models—ANN, CNN, RNN, and LSTM. Each model is represented by a distinct color, with data points scattered around the diagonal line representing ideal predictions where actual and predicted values would align perfectly. The dense clustering of points near the diagonal suggests that most models are capable of reasonably accurate predictions. However, variations and dispersions, particularly at higher output levels, indicate areas where the models differ in predictive accuracy. Overall, Fig 17 highlights ANN’s tighter clustering along the trend line, confirming its robustness compared to other models.

### 3.3. Machine learning vs. deep learning comparison for wind turbine prediction

Table 6 presents a comparative examination of ML and DL models for forecasting WT power production. The ANN attained the best accuracy (R^2^: 0.7248) and the lowest RMSE (0.1516), closely succeeded by the ET model (R^2^: 0.7231, RMSE: 0.1512). ML ensemble methods like RF and CatBoost also demonstrated strong performance, while simpler ML models like LR and AdaBoost lagged. DL models like RNN and LSTM showed moderate accuracy, while CNN underperformed slightly, with minimal differences. Table 6 highlights ANN’s superior accuracy and ML ensembles’ computational efficiency, providing insights for model selection based on use-case priorities.

**Table 6:** Summary of comparative analysis between ML and DL models for predicting WT power output.

Aspect	ML Models	DL Models	Key Insights
**Top Performers**	ET (R^2^: 0.7231) RF (R^2^: 0.7185)	ANN (R^2^: 0.7248) RNN (R^2^: 0.6551)	ANN and ET performed best.
**Predictive Accuracy (R^2^)**	High for ensemble models (ET, RF)	Best for ANN; moderate for RNN and LSTM	ANN and ML ensembles are comparable.
**Error Metrics**	Lowest RMSE: ET (0.1512), RF (0.1525)	Lowest RMSE: ANN (0.1516)	Similar errors for ANN and ET.
**Computational Complexity**	Moderate for ensemble models	High for ANN, LSTM, RNN	ML is more efficient than DL.
**Generalization**	Excellent for ensembles, moderate for simpler ML	Excellent for ANN, moderate for CNN	ANN and ML ensembles generalize well.
**Suitability**	Best for structured data, moderate nonlinearity	Best for nonlinear and sequential data	ML is practical for efficiency; DL for complexity.

## 4. Conclusion

In this study, a detailed comparative analysis of ML and DL models has been successfully carried out for WT power output prediction using environmental variables. After modeling eight ML models—LR, SVR, RF, ET, AdaBoost, CatBoost, XGBoost, and LightGBM—as well as four DL models—ANN, LSTM, RNN, and CNN—the research found that ANN outperformed the rest, with the best R^2^ (0.7248) and lowest RMSE (0.1516), with ET coming up very close as the top ML model. In this work, the study presented a novel direct comparison of various ML and DL approaches to highlight their novelty in capturing the nonlinear relationships that exist between environmental factors and WT power output. Highlighting the importance of preprocessing and hyperparameter optimization in boosting predictive accuracy, the research showed how advanced computational approaches are able to transform renewable energy forecasting and operational optimization.

Future studies should further test these findings using additional environmental variables, including atmospheric pressure and solar radiation, to both improve model precision and generalizability. Lastly, the exploration of hybrid models which combine ML and DL techniques reveals new opportunities for improving predictive capability. A more robust assessment of these models adaptability will be possible across different geographic regions and under varied climatic conditions. Additionally, investigations into how predictable their performance is would improve their integration with predictive models in real time and in WT control systems, as well as the resilience of the expected performance for dynamic climate change. The optimization of renewable energy systems would be so greatly advanced that they contribute to overall global sustainability goals. Moreover, K-Means or hierarchical clustering could have been used to form these environmental conditions into clusters based on common characteristics. These clusters can also enable insights into different operational regimes that affect WT power output. Furthermore, integrating cluster-based modeling would improve prediction accuracy by training ML models on an environmental pattern.

## Supporting information

S1 Data Data in the experiment. (ZIP)

## Abbreviations

AdaBoost: Adaptive Boosting
ANN: Artificial Neural Networks
CatBoost: Categorical Boosting
CNN: Convolutional Neural Network
CN: Carbon Neutral
DL: Deep Learning
DWPT: Dew Point
EDA: Exploratory Data Analysis
ET: Extra Trees
LightGBM: Light Gradient Boosting Machine
LR: Linear Regression
LSTM: Long Short-Term Memory
MAE: Mean Absolute Error
ML: Machine Learning
PV: Photovoltaic
RF: Random Forest
RES: Renewable Energy Sources
RH: Relative Humidity
RMSE: Root Mean Square Error
RNN: Recurrent Neural Network
SVR: Support Vector Regressor
WT: Wind Turbine
XGBoost: Extreme Gradient Boosting
